# Identification of Novel Transcribed Regions in Zebrafish (*Danio rerio*) Using RNA-Sequencing

**DOI:** 10.1371/journal.pone.0160197

**Published:** 2016-07-27

**Authors:** Jingwen Wang, Liselotte Vesterlund, Juha Kere, Hong Jiao

**Affiliations:** 1 Department of Biosciences and Nutrition, Science for Life Laboratory, Karolinska Institutet, Stockholm, Sweden; 2 Clinical Research Centre, Karolinska University Hospital, Huddinge, Sweden; 3 Department of Medical Genetics, Hartman Institute, University of Helsinki, Finland; 4 Folkhälsan Institute of Genetics, Helsinki, Finland; Institut National de la Recherche Agronomique (INRA), FRANCE

## Abstract

Zebrafish (*Danio rerio*) has emerged as a model organism to investigate vertebrate development and human genetic diseases. However, the zebrafish genome annotation is still ongoing and incomplete, and there are still new gene transcripts to be found. With the introduction of massive parallel sequencing, whole transcriptome studies became possible. In the present study, we aimed to discover novel transcribed regions (NTRs) using developmental transcriptome data from RNA sequencing. In order to achieve this, we developed an in-house bioinformatics pipeline for NTR discovery. Using the pipeline, we detected 152 putative NTRs that at the time of discovery were not annotated in Ensembl and NCBI gene database. Four randomly selected NTRs were successfully validated using RT-PCR, and expression profiles of 10 randomly selected NTRs were evaluated using qRT-PCR. The identification of these 152 NTRs provide new information for zebrafish genome annotation as well as new candidates for studies of zebrafish gene function.

## Introduction

Transcriptome analysis has become a key tool for understanding functional roles of genes involved in a variety of biological processes including early development [1]. In 2005 Mathavan et al. [2] published a genome-wide microarray analysis of the embryonic zebrafish transcriptome using 12 different embryonic time points. The study revealed a highly dynamic and diverse transcriptional profile during embryogenesis and identified a previously unknown set of very early genes transcribed prior to the mid-blastula transition (MBT). Over the last decade, technologies for transcriptome analysis have improved dramatically, and high throughput RNA sequencing technologies (RNA-Seq) have revolutionized transcriptomics by detailed examination of cellular transcriptomes with high sensitivity, high dynamic range, and expression at a single-cell resolution [3, 4]. Unlike microarray expression analysis, transcriptome profiling using RNA-Seq may allow for discovery of novel transcribed regions (NTRs) since it is not limited by the availability of reference information.

Zebrafish is a valuable vertebrate model organism for studies in developmental biology [5] and for functional characterization of human disease genes, especially where the functional tissue such as brain is not readily available in human [6]. However, the zebrafish genome annotation is not complete and still ongoing. Therefore, zebrafish sequencing data provide an opportunity for NTR discovery. Several whole transcriptome analyses using RNA-Seq have been performed in zebrafish [7–11]. Besides unraveling expression profiles of transcriptome dynamics during early embryonic stages, a number of NTRs in annotated and unannotated regions of the zebrafish genome have also been described [7, 8, 12]. The aim of this study is to discover novel transcribed regions (NTRs) using developmental transcriptome data from RNA sequencing. We have identified 152 putative NTRs that at the time of discovery were not annotated in Ensembl and NCBI gene database using an in-house bioinformatics pipeline to systematically in zebrafish early development by reanalyzing our previously obtained RNA-Seq data [8].

## Materials and Methods

### RNA preparation and sequencing

Embryo collection, RNA extraction and RNA-Seq have been described previously [8]. All sequence data (accession numbers ERP000635 and PRJEB9889) are available at the European Nucleotide Archive (http://www.ebi.ac.uk/ena/) website. In summary, total RNA was extracted from four developmental stages (1-cell (0.75 hpf), 16-cell (1.5 hpf), 512-cells (2.75 hpf) and 50% epiboly (5.25 hpf)) and subsequently rRNA depleted using RiboMinus^**™**^ Eukaryote Kit for RNA-Seq (Life Technologies). We performed in total three runs of RNA-Seq on the SOLiD System Sequencing platform (ABI, Applied Biosystems), with two biological replicate runs and one technical replicate run. The libraries were sequenced using 50 base pair (bp) reads. The research protocol was approved by the local Ethical Board, Stockholms Norra Djurförsöksetiska Nämnd (application number N413/11, N230/10 and S170/08).

### Reads mapping and filtering

RNA-Seq data were analyzed using Tophat v2.0.4 [13] that applies Bowtie v.0.12.8 to handle color space reads generated by the SOLiD (ABI, Applied Biosystems). Briefly, after quality check [8] the sequencing reads from three runs at four stages were individually aligned to the zebrafish reference genome danRer7/Zv9 assembly from Ensembl. Only uniquely mapped reads were used for NTR discovery. Read filtering was performed using SAMtools [14].

### Detection of putative NTRs

**Fig 1:**
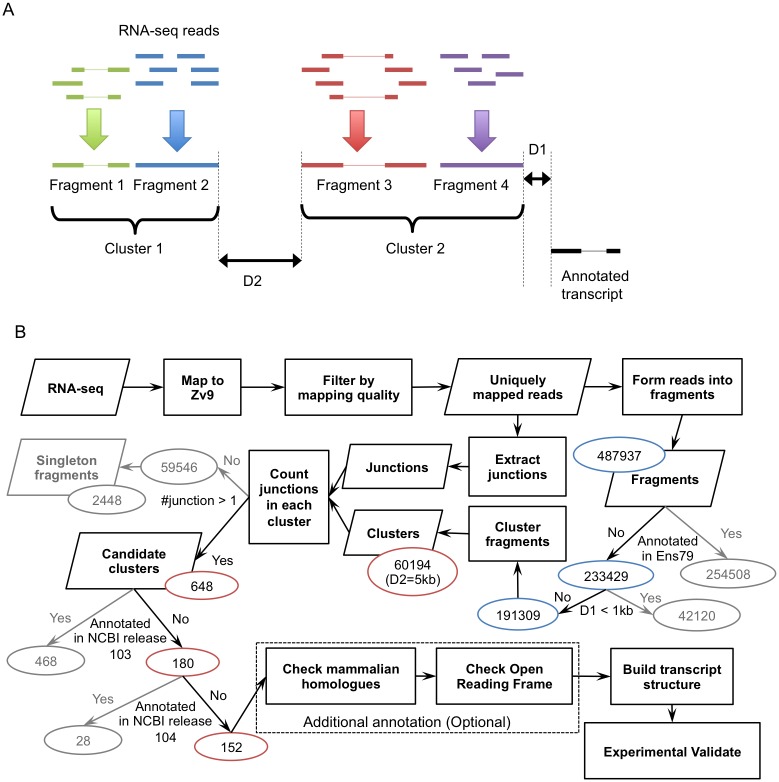
Systematic workflow for the identification of NTRs using RNA-Seq datasets. A. RNA-Seq reads form fragments (shown in green, blue, red and purple), which further generate clusters for NTR identification. D1: the distance between a putative NTR and any annotated transcribed regions; D2: the distance between two putative NTRs; B. Systematic workflow of NTR identification. The numbers for fragments are circled in blue, while numbers for clusters are in red circles. Singleton fragments are one-fragment clusters with length over 50 bp.

NTRs were detected using in-house pipeline composed of BEDTools [15] modules (mergeBed, slopBed, intersectBed) and customized scripts. The pipeline conducts two types of tasks, fragment construction based on the uniquely mapped RNA-Seq reads and formation of clusters (Fig 1). A fragment is defined as a number of adjacent reads overlapped by one or more bp at ends on the same strand or a read with detected splicing junction sites. A cluster is a group of linked fragments on the same strand. Output of the pipeline could be controlled by two parameters, D1 and D2. D1 is the distance between a cluster and any annotated transcript, while D2 is the distance between two adjacent clusters (Fig 1A).

In order to find independent NTRs, fragments with a distance D1 away from any known transcripts on the same strand at either end were excluded from clustering since they could be additional parts of those known transcripts. Ensembl genome annotation release 79 was used as a primary reference to detect putative NTRs. In addition, we also checked all detected NTRs against NCBI zebrafish (*Danio rerio*) annotation releases 103 and 104 for further known transcript filtering.

For clustering, any two fragments with a distance less than D2 on the same strand were considered as linked. A series of linked fragments formed a cluster. A cluster could be defined as a putative NTR if 1) it included at least two splicing junction sites detected by TopHat; 2) it was not annotated in the databases mentioned above.

### *In silico* confirmation of putative NTRs

A variety of *in silico* methods were used to confirm the presence of putative NTRs. Besides using junction information to clarify hypothetical exon boundaries within clusters, we also used gene models provided by GENSCAN [16] and open reading frame (ORF) predicted using FGENESH software (http://www.softberry.com) to confirm the structure of potential protein-coding genes. Zebrafish CAGE data [11] were used as additional supporting evidence. Moreover, we also looked for conservation of NTRs using human proteins mapped by tBLASTn and RefSeq gene of other species, which were download from the UCSC genome browser (http://hgdownload.soe.ucsc.edu/goldenPath/danRer7/database/xenoRefGene.txt.gz, http://hgdownload.soe.ucsc.edu/goldenPath/danRer7/database/blastHg18KG.txt.gz).

### Experimental validation of putative NTRs

Total RNA was extracted from zebrafish embryos at different developmental stages using Trireagent (Sigma Aldrich). cDNA was synthesized using SuperScript III First-Strand Synthesis SuperMix for reverse transcription polymerase chain reaction (RT-PCR) according to the manufacturer’s protocol (Invitrogen, Life Technologies). Half a microgram of the total RNA was taken as input material. HotStarTaq plus DNA polymerase (Qiagen) was used in combination with specific PCR primers (S1 Table) to amplify regions of the putative transcripts. The fragments were subsequently cloned into pCRII-TOPO vector (Invitrogen, Life Technologies) and validated using Sanger sequencing (Eurofin Genomics, Ebersberg, Germany). cDNA from 50% epiboly was used as template. The primer for validation was designed by using Primer3 [17, 18], and they were located at the first and the last exons of each predicted isoform.

### Expression evaluation of putative NTRs

The expression profiles of the discovered 152 NTRs were first evaluated based on the RNA-Seq from the biological replicate samples at all four studied developmental stages. The Cuffdiff module from Cufflinks 2.0.0 [19] was employed to estimate expression levels in terms of fragments per kilobase of transcript per million mapped reads (FPKM). Gplots package in R (http://CRAN.R-project.org/package=gplots) was employed to explore the expression profiles of the 152 NTRs. The expression levels (FPKM) were scaled in each NTR using heatmap2 function. Subsequently, 10 NTRs were randomly selected for validation using quantitative reverse transcription polymerase chain reaction (qRT-PCR).

For the qRT-PCR validation, RNA extraction and cDNA synthesis were performed as described above. The developmental stages used in cDNA synthesis were 1-cell, 16-cell, 512-cell and 50% epiboly. The primers (S1 Table) were designed to span the exon splicing junctions, 150–200 bp in length, by using Primer3 [17, 18]. The gene expression analysis was performed in duplicates on three biological replicate samples of these four developmental stages. Expression of bactin2 in each developmental stage was used as the control. Fast SYBR Green Master Mix (Thermo Fisher Scientific) was used for qRT-PCR according to manufacturers protocol and the experiments were run on the Applied Biosystems 7500 Real-Time PCR system. All amplicons were validated using Sanger sequencing.

## Results

### RNA-Seq data mapping

Each of the three runs of previously obtained RNA-Seq data from four zebrafish embryonic developmental stages (1-cell, 16-cell, 512-cell and 50% epiboly) [8] was realigned individually using TopHat v2.0.4 [13] against the zebrafish reference genome Zv9. The realignment was done using TopHat default parameters corresponding to SOLiD sequencing data and no genome annotation was used. In total, we obtained more than 200 million raw sequencing reads for each developmental stage from all three runs. Table 1 shows the raw read amounts of the four developmental stages in each run and the mapping rates for each stage based on the total read numbers. About 62–76 million reads could be mapped to the zebrafish reference genome Zv9, accounting for 26%-34% of the total reads from different stages. At each developmental stage, 37–55 million reads were uniquely mapped and subsequently used for NTR discovery.

**Table 1:** Read counts (in million) in four developmental stages.

	1-cell	16-cell	512-cell	50% epiboly
**Run 1 (original)**	114,4	105,6	109,1	106,6
**Run 2 (technical replicate)**	52,5	54,4	40,6	50,8
**Run 3 (biological replicate)**	73,5	85,1	78,4	78,6
*Total reads	240,4	245,1	228,0	235,9
**Mapped reads**	61,9	64,4	76,3	60,6
**Mapped reads (%)	25,8	26,3	33,5	25,7
**Uniquely mapped reads**	38,0	39,1	52,9	36,0

*A sum of reads in million from all three runs at corresponding developmental stages.

**Proportion against to the total reads for each developmental stage.

### Discovery of NTRs

The workflow of NTR discovery is described in Fig 1B. In order to increase the read coverage and depth, we pooled uniquely mapping reads from all four stages and all three runs. With the pooled reads (about 166 million in total) as the input, we obtained 487937 fragments with either overlapped reads and/or reads containing splicing junction sites. Among them, 254508 fragments were located in annotated transcripts regions (Ensembl genome annotation release 79). To avoid potential extensions of annotated transcripts, we further filtered out 42120 fragments with D1 ≤ 1 kb, i.e. 1 kb or less away from any known transcripts. The remaining 191309 fragments formed 60194 clusters with separating distances less or equal to 5 kb between fragments (D2 to ≤ 5kb). We calculated numbers of splicing junction within each cluster and excluded those clusters without or having only one splicing junction site to avoid potential random error (Fig 1B). After this filtering step, we obtained 648 NTRs (S2 Table). Among the 648 NTRs, 449 were predicted by GENSCAN and 105 had putative homologues in mammals.

**Fig 2:**
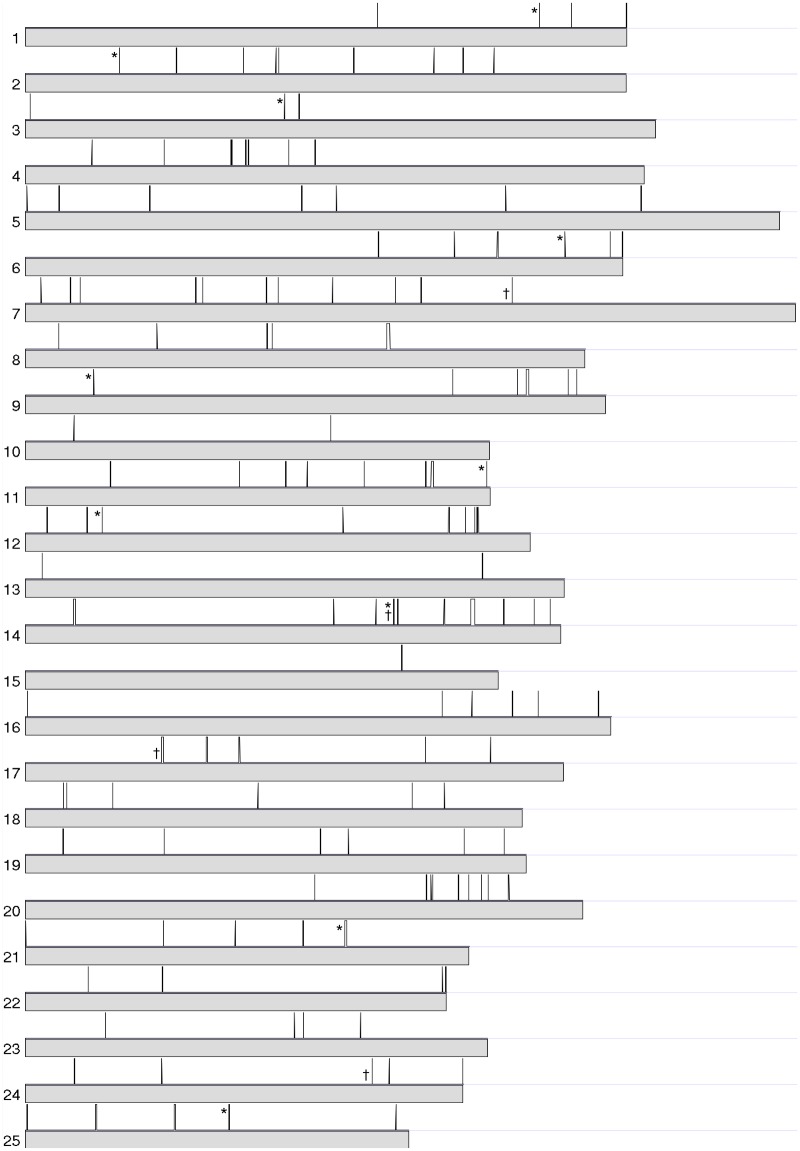
Position of discovered NTRs in the zebrafish genome. The USCS Zebrafish Genome Graphs tool was used to generate the figure. The 152 NTRs with approximate genomic positions (vertical lines in black) were detected on each chromosome (represented by grey bars) of the zebrafish genome. The NTRs marked with crosses were validated using RT-PCR, and those marked with asterisks were evaluated using qRT-PCR.

During the course of the study, the zebrafish genome annotations have been continuously updated. Therefore, some of the NTRs predicted based on Ensembl genome annotation release 79 were subsequently annotated in NCBI zebrafish annotation release 103, validating our approach for those genes. However, even with the exclusion of these newly annotated regions, there were 180 NTRs remaining unannotated (Fig 1B). Among those NTRs, 28 were annotated in NCBI zebrafish annotation release 104 (Zv10 zebrafish reference genome) currently. Therefore, as a final result, we have identified 152 NTRs that had not been previously annotated in the databases (Table 2). These NTRs are distributed on all 25 chromosomes in the zebrafish genome (Fig 2).

**Table 2:** Putative NTRs without annotation in NCBI Zebrafish Annotation.

ID	Chr	Start*	End*	Read	Fragment	Strand	Junctions	ORF#	CAGE [10]§
**NTR1**	1	35319661	35339274	45	9	+	3	No	No
**NTR2**	1	51577333	51594446	2298	15	-	2	Yes	No
**NTR3**	1	54766456	54769088	58	1	-	2	No	No
**NTR4**	1	60296527	60303254	186	13	-	2	No	No
**NTR5**	2	9452513	9457753	152	6	-	5	No	No
**NTR6**	2	9456965	9465868	1617	15	+	5	Yes	No
**NTR7**	2	15129192	15195034	14	1	+	3	No	No
**NTR8**	2	21906509	21909596	1973	2	-	4	No	Yes
**NTR9**	2	25134495	25164990	35	9	-	3	No	No
**NTR10**	2	25456315	25457120	769	3	-	2	No	No
**NTR11**	2	32935627	32997999	35	10	+	2	No	No
**NTR12**	2	40984882	40994077	61	7	-	2	No	No
**NTR13**	2	43909722	43923665	17	10	+	2	No	No
**NTR14**	2	46995468	47021635	112	24	-	2	No	No
**NTR15**	3	483190	499376	91	17	-	2	Yes	No
**NTR16**	3	26008735	26014066	1268	3	+	2	No	No
**NTR17**	3	27445420	27506280	118	13	+	3	Yes	No
**NTR18**	4	6689773	6722999	199	8	+	2	No	No
**NTR19**	4	13930584	13954529	21	2	-	2	No	No
**NTR20**	4	20640325	20641139	3560	1	-	2	No	No
**NTR21**	4	20690290	20731953	20	3	+	2	No	No
**NTR22**	4	22083568	22141712	71	16	-	2	No	No
**NTR23**	4	22384549	22397950	64	10	+	2	No	No
**NTR24**	4	26450122	26451848	44	12	-	2	No	No
**NTR25**	4	29070796	29103409	428	40	+	3	Yes	No
**NTR26**	5	177435	190646	317	7	-	2	Yes	No
**NTR27**	5	3407117	3468535	569	9	-	4	Yes	No
**NTR28**	5	12440480	12500047	478	15	-	4	Yes	No
**NTR29**	5	27729893	27758811	16	8	+	2	No	No
**NTR30**	5	31199261	31213579	29	14	+	2	No	No
**NTR31**	5	48164838	48194517	43	1	-	2	Yes	No
**NTR32**	5	61742376	61782718	1502	26	+	2	Yes	No
**NTR33**	6	35425230	35438221	174	6	-	2	No	No
**NTR34**	6	43025348	43045695	6	3	-	2	No	No
**NTR35**	6	47336421	47413477	88	1	-	3	No	No
**NTR36**	6	54121395	54141521	104	42	+	2	Yes	No
**NTR37**	6	58664365	58672163	4	2	+	2	No	No
**NTR38**	6	59879110	59902431	64	2	+	2	Yes	No
**NTR39**	7	1591529	1592856	286	4	+	2	No	No
**NTR40**	7	4530486	4572786	81	39	-	2	No	No
**NTR41**	7	5527626	5537794	112	36	-	2	No	No
**NTR42**	7	17101078	17114877	502	12	-	2	No	No
**NTR43**	7	17798541	17801728	50	2	+	2	No	No
**NTR44**	7	24188830	24209179	40	7	+	2	No	No
**NTR45**	7	25377119	25377706	78	2	+	2	No	No
**NTR46**	7	30826740	30836802	12	3	-	3	No	No
**NTR47**	7	37119991	37122678	20	6	+	2	No	No
**NTR48**	7	37129478	37147767	156	5	+	3	No	No
**NTR49**	7	39701577	39738113	514	47	+	2	No	No
**NTR50**	7	48846088	48859085	2276	4	-	5	No	No
**NTR51**	8	3366664	3376896	33	4	-	2	No	No
**NTR52**	8	13213857	13238686	5	2	-	2	Yes	No
**NTR53**	8	24259885	24275933	75	24	+	2	No	No
**NTR54**	8	24751655	24762798	30	8	+	2	No	No
**NTR55**	8	36298679	36566230	1798	2	-	3	Yes	No
**NTR56**	8	36446071	36461541	211	7	+	2	Yes	No
**NTR57**	9	6834008	6880805	1129	5	-	2	Yes	No
**NTR58**	9	42861451	42879578	18	7	+	2	No	No
**NTR59**	9	49346760	49349349	9	5	+	2	No	No
**NTR60**	9	50252952	50489824	146	1	-	3	No	No
**NTR61**	9	54434803	54435791	85	1	+	2	No	No
**NTR62**	9	55311024	55333808	136	6	-	2	No	No
**NTR63**	10	4884897	4928547	41	9	-	2	No	No
**NTR64**	10	30632959	30642845	22	3	+	2	No	No
**NTR65**	10	30652656	30654363	81	10	+	2	No	No
**NTR66**	11	8555439	8604737	72	12	-	3	Yes	No
**NTR67**	11	21471757	21498121	10	2	-	2	No	No
**NTR68**	11	26114943	26144022	24	14	+	2	No	No
**NTR69**	11	28280836	28292899	44	6	+	2	No	No
**NTR70**	11	34000672	34017832	347	5	-	2	Yes	No
**NTR71**	11	40166959	40180327	19	3	+	2	Yes	No
**NTR72**	11	40710652	40916338	102	3	+	2	No	No
**NTR73**	11	46299240	46305531	728	10	+	2	Yes	No
**NTR74**	12	2153998	2212771	187	24	+	4	Yes	No
**NTR75**	12	6213398	6238582	78	4	+	2	No	No
**NTR76**	12	7712953	7734679	347	24	-	2	No	No
**NTR77**	12	31868771	31880619	42	3	-	2	No	No
**NTR78**	12	42457304	42551873	67	6	-	3	Yes	No
**NTR79**	12	44135425	44149684	228	58	+	3	Yes	No
**NTR80**	12	45074145	45090687	53	8	-	4	Yes	No
**NTR81**	12	45302279	45303716	30	3	+	2	No	No
**NTR82**	12	45387801	45453816	60	4	-	2	No	No
**NTR83**	13	1684265	1698909	39	3	-	2	No	No
**NTR84**	13	45819938	45875603	58	2	-	3	Yes	No
**NTR85**	14	4857007	5066829	24	3	-	2	No	No
**NTR86**	14	30927030	30949516	98	12	+	2	No	No
**NTR87**	14	35161104	35212736	153	3	-	2	No	No
**NTR88**	14	36936135	37018037	110	6	-	3	No	Yes
**NTR89**	14	37318303	37391089	349	14	-	3	No	No
**NTR90**	14	41988399	42050638	200	17	-	4	Yes	No
**NTR91**	14	44714986	45097038	8307	1	+	26	No	Yes
**NTR92**	14	47993927	48003335	576	3	+	3	Yes	No
**NTR93**	14	51046773	51056639	1313	2	-	3	No	No
**NTR94**	14	52658942	52666335	1881	27	+	2	No	No
**NTR95**	15	37755792	37826124	264	61	-	6	No	No
**NTR96**	16	212149	214655	254	3	+	2	No	No
**NTR97**	16	41821609	41827843	82	4	-	2	No	No
**NTR98**	16	44796081	44803583	30	4	-	2	Yes	No
**NTR99**	16	48851091	48879000	141	20	+	2	No	No
**NTR100**	16	48895012	48899918	6	3	+	2	No	No
**NTR101**	16	51420049	51435991	27	7	-	2	Yes	No
**NTR102**	16	57446118	57503283	922	9	-	2	No	No
**NTR103**	17	13676981	13889276	570	10	+	4	No	No
**NTR104**	17	18140319	18252834	59	8	+	3	No	No
**NTR105**	17	21456232	21513529	51	16	+	2	No	No
**NTR106**	17	40130880	40163594	33	8	-	2	No	No
**NTR107**	17	46669464	46672104	203	1	-	2	No	No
**NTR108**	18	3825791	3833813	63	6	-	2	Yes	No
**NTR109**	18	4178878	4191305	5	3	-	2	No	No
**NTR110**	18	8776376	8777440	62	1	+	3	No	No
**NTR111**	18	23336289	23379195	242	2	-	3	Yes	No
**NTR112**	18	38809397	38818932	102	11	-	2	Yes	No
**NTR113**	18	42038290	42049662	34	8	+	2	No	No
**NTR114**	19	3796532	3839885	114	7	+	3	No	No
**NTR115**	19	13939843	13945375	24	8	+	2	No	No
**NTR116**	19	29590118	29611600	84	19	-	2	No	No
**NTR117**	19	32384216	32418038	43	14	+	2	No	No
**NTR118**	19	44026717	44037083	143	4	+	2	No	No
**NTR119**	19	48035337	48039154	9	3	-	2	Yes	No
**NTR120**	20	29053976	29067865	75	8	+	4	No	No
**NTR121**	20	40232343	40255853	116	10	-	2	No	No
**NTR122**	20	40698114	40702888	32	3	+	4	No	No
**NTR123**	20	40873906	40879859	20	9	+	2	Yes	No
**NTR124**	20	43442015	43454643	16	9	+	2	No	No
**NTR125**	20	44479515	44484380	29	4	-	2	Yes	No
**NTR126**	20	45742830	45755594	40	9	-	2	No	No
**NTR127**	20	46421708	46425130	10	3	-	2	No	No
**NTR128**	20	48450259	48523788	51	2	-	2	No	No
**NTR129**	21	45360	62133	138	22	-	3	No	No
**NTR130**	21	13859367	13885087	75	2	+	4	No	No
**NTR131**	21	21061627	21063121	9	4	+	2	No	No
**NTR132**	21	27837726	27885625	190	71	-	6	No	No
**NTR133**	21	32065948	32154412	187	10	+	3	Yes	No
**NTR134**	21	32161868	32267827	146	6	-	2	No	No
**NTR135**	22	6291894	6303009	16	4	-	2	No	No
**NTR136**	22	13738611	13781435	104	19	-	3	No	No
**NTR137**	22	41805942	41840524	107	26	-	2	No	No
**NTR138**	22	42174880	42214019	284	45	-	7	Yes	No
**NTR139**	23	8038211	8060627	58	3	-	4	Yes	No
**NTR140**	23	26967897	27002811	151	23	-	3	Yes	No
**NTR141**	23	27890549	27902389	119	12	-	2	No	No
**NTR142**	23	33622328	33658841	105	20	-	2	No	No
**NTR143**	24	4938353	4965187	42	3	-	2	Yes	No
**NTR144**	24	13658228	13694444	20	3	+	2	No	No
**NTR145**	24	34773520	34793936	205	8	+	4	Yes	No
**NTR146**	24	36502404	36543849	57	37	+	2	Yes	No
**NTR147**	24	43894672	43896091	88	4	+	3	No	No
**NTR148**	25	198510	222254	36	1	-	2	Yes	No
**NTR149**	25	7084534	7166975	47	7	+	2	Yes	Yes
**NTR150**	25	14954933	15041945	367	9	-	3	No	No
**NTR151**	25	20454575	20479050	99	12	-	2	No	No
**NTR152**	25	37176375	37181295	75	1	-	2	Yes	No

* The start and end position were defined according to the coverage of RNA-Seq reads.

^#^ORF prediction was performed with GENSCAN program.

^§^ The NTR was marked as "Yes" if there were reads covered in the promoter region according to zebrafish CAGE data.

### Validation of putative NTRs

Before experimental validation, structures of NTRs built based on RNA-Seq data were checked *in silico* to investigate overlap with any predicted open reading frames (ORF) or potential gene models. The expression of NTRs was validated using RT-PCR with pooled RNA samples from 50% epiboly. We used Sanger sequencing to confirm the sequences of amplified PCR products.

**Fig 3:**
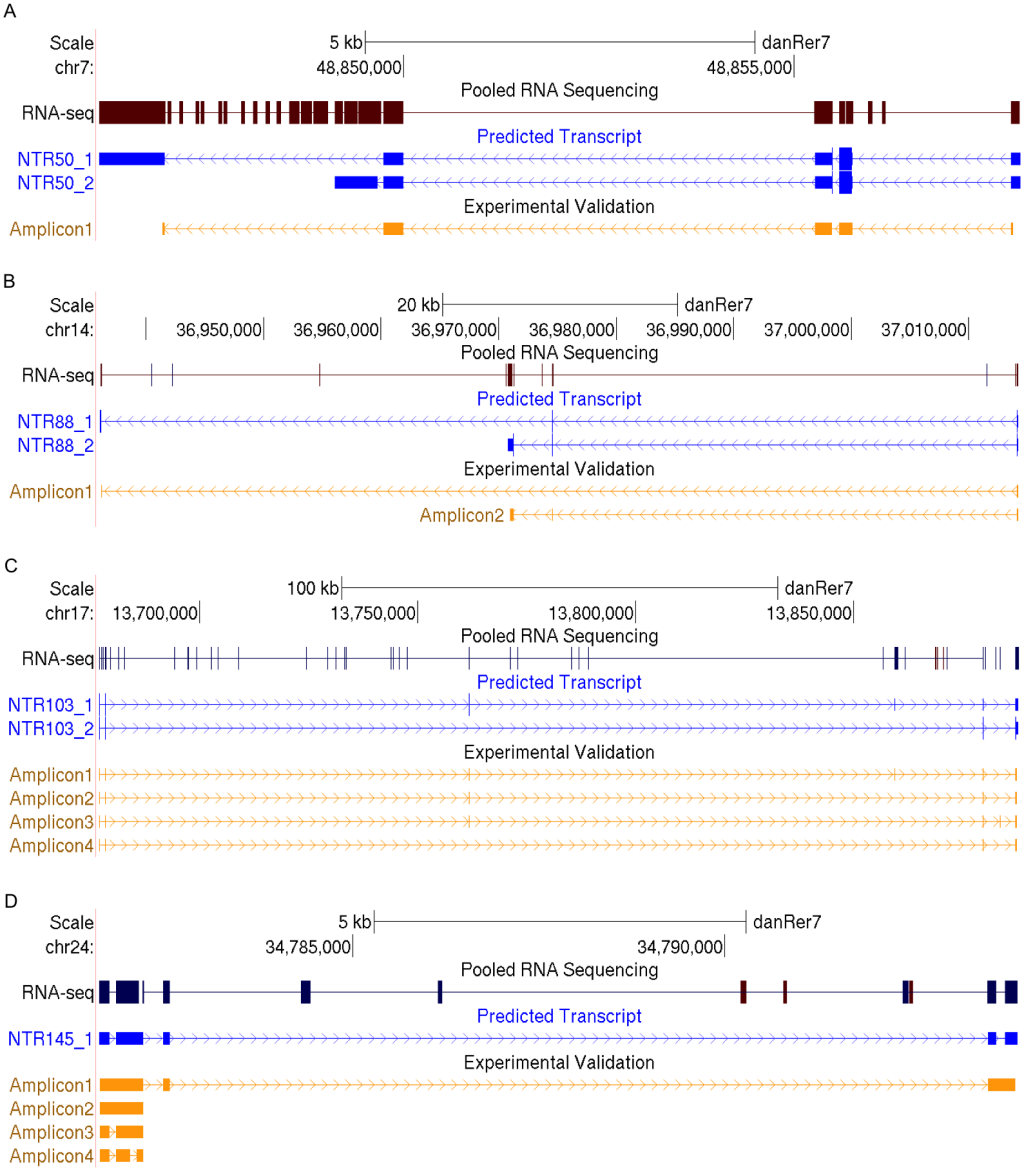
Prediction and validation of 4 randomly selected NTRs. The RNA samples used for validation were extracted from 50% epiboly. RNA-seq: RNA-Seq tracks (red boxes) based on the pooled RNA-Seq data; NTRs: Predicted structures of putative NTRs by our pipeline. Blue boxes represent fragments; Amplicons: Validations of the predicted NTR structures by RT-PCR. “Chr” indicates chromosome. A. NTR50; B. NTR88; C. NTR103; and D. NTR145.

Four NTRs, NTR50, NTR88, NTR103, and NTR145 were randomly selected for experimental validation. In general, the validation results showed high similarity with the predicted structures of NTRs (Fig 3). One isoform of NTR50, NTR50_1, was validated as amplicon1 in Fig 3A. We were not able to detect the predicted isoform NTR50_2 in 50% epiboly. Sanger sequencing confirmed both isoforms of NTR88 (Fig 3B), in agreement with the predicted structures. The two isoforms were supported by ORF prediction as well. For NTR103, besides two predicted isoforms being confirmed, we also detected two additional isoforms, amplicon2 and amplicon3 (Fig 3C). For NTR145, we found three additional alternative splicing patterns at the 5’-end of the NTR (Fig 3D).

### Expression of NTRs

The expression of the discovered 152 NTRs was evaluated using RNA-Seq data from the biological replicates run using the Cufflinks. Cuffdiff module. S1 Fig shows the expression profiles of these 152 NTRs. Except for NTR20 and NTR10 that were highly expressed in 50% epiboly, all the other NTRs showed relatively low expression levels (FPKM ≤ 10) in the four studied early developmental stages. The average expression values were below 1 FPKM for more than 90% of the NTRs. Five NTRs (NTR8, NTR41, NTR61, NTR81 and NTR115) were upregulated at 50% epiboly by more than 1 FPKM compared with the 512-cell stage (S2A Fig). Several other NTRs also demonstrated a clear upregulated pattern after MBT albeit at lower expression levels (S2B Fig). Many NTRs showed a peak in expression levels at the 512-cell stage (S2D, S2E and S2F Fig). Out of the six selected expression patterns, two displayed relatively high expression values (S2A and S2D Fig). The NTRs displaying an increase after MBT (NTR61, NTR81 and NTR115) may be associated with processes important for organismal and anatomical structure development (S2A Fig) [7, 8]. The NTRs showing relatively high maternal expression were expressed throughout MBT to subsequently diminish in 50% epiboly stage (S2D Fig).

**Fig 4:**
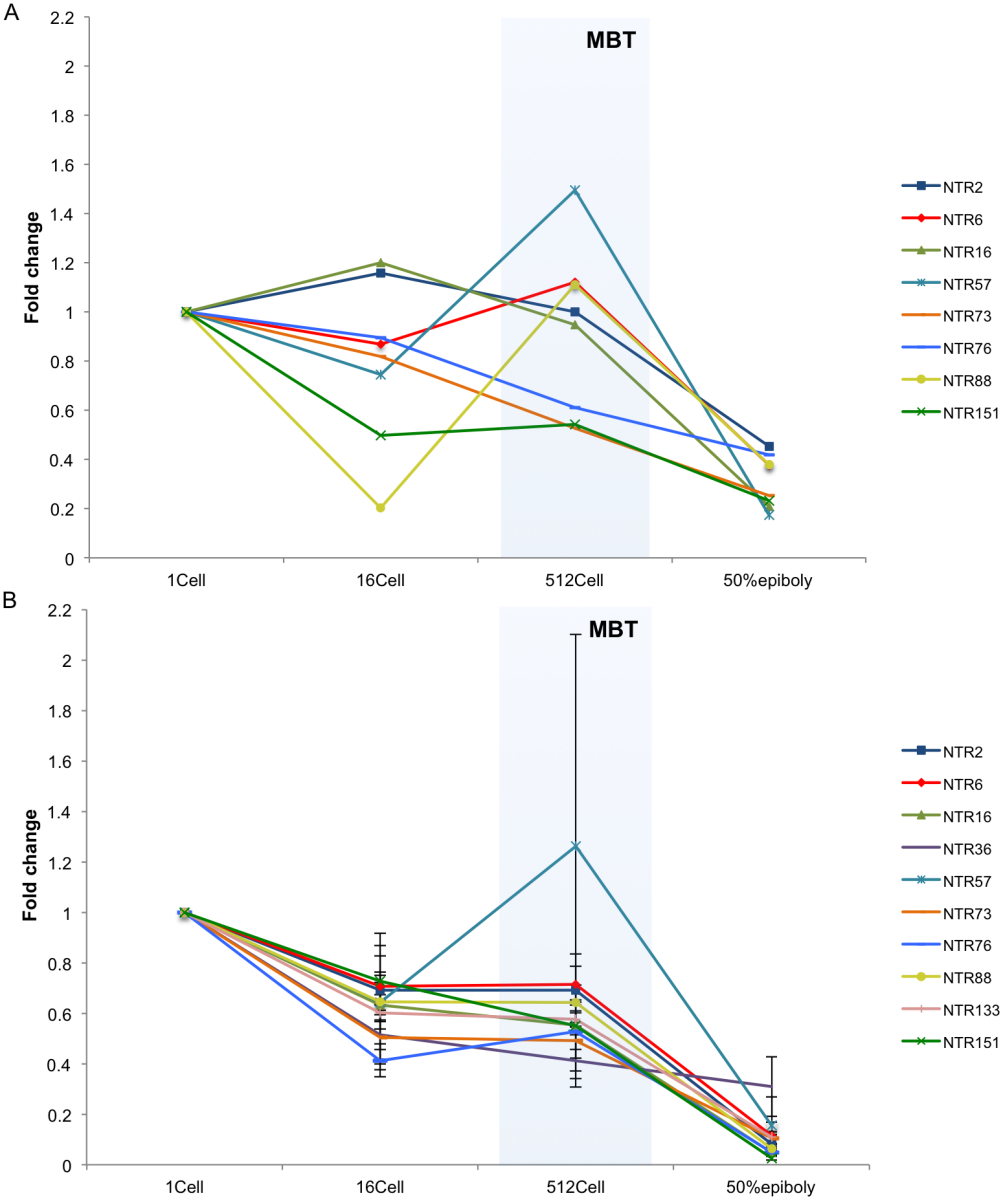
Gene expression profiles of 10 randomly selected NTRs. A. The upper panel shows the gene expression fold change of the randomly selected 10 NTRs calculated from RNA-Seq data, except NTR 36 whose fold change was over 29 at 50% epiboly, and NTR133 which was not expressed at 1-cell stage; B. The lower panel shows the gene expression fold changes of the 10 randomly selected NTRs calculated from qRT-PCR. The 1-cell stage was used as basal line for fold change calculations. Developmental stages are given on the X-axis. MBT—Midblastula transition.

We randomly selected 10 NTRs for gene expression profile validation. According to our RNA-Seq data analysis, 8 out of the 10 NTRs were downregulated as the embryo development progressed from 1-cell to 50% epiboly (Fig 4A). Two exceptions were NTR36, with expression level showing 30-fold change from 1-cell to 50% epiboly, and NTR133, which was only detected as expressed at 50% epiboly. All 10 NTRs were validated using qRT-PCR (S3 Fig). The expression levels of NTR36, NTR57, NTR76, NTR88 and NTR151 were low at all studied stages (S4 Fig). The expression measured by qRT-PCR replicated a similar expression profile for the 8 downregulated NTRs (Fig 4B). However, both NTR36 and NTR133 were detected as downregulated at the 50% epiboly stage by qRT-PCR.

## Discussion

### RNA-Seq analysis and pipeline development

In our previous study [8] we used Bioscope that is developed specifically for color space reads from the Applied Biosystems Inc. (ABI). Bioscope could provide 65–73% mapping rates. However, it provides junction information only for known transcripts, not for unannotated regions. Thus, for NTR discovery, we chose TopHat because it is capable of detecting novel splice sites, even though having a lower mapping rate compared with Bioscope. TopHat uses an efficient read-mapping algorithm designed to align reads from a RNA-Seq experiment to a reference genome without relying on known splice sites [13].

For NTR discovery, we did not use Cufflinks package that focuses on finding new isoforms of annotated transcripts as a previous study did [12]. In our study, we aimed to find NTRs that were not annotated. Although Cufflinks could provide information of transcribed fragments in intragenic regions, the program would not consider the separated fragments as linked transcript even if they are located close by to each other without overlapping. Our pipeline is more flexible handling these fragments. It assesses those reads with adjustable parameters D1 and D2 (Fig 1A). The potential random errors will be restricted by numbers of splicing junction in a putative NTR and other predication methods.

There are two input parameters, D1 and D2, in our pipeline for NTR discovery (Fig 1A). The values we used for D1 and D2 were arbitrary. D1 was used to avoid potential extension to an annotated gene by additional exon/s. We set D1 to 1 kb, so that any fragments located at most 1 kb away from any known transcripts were excluded. D1 could be increased to reduce the probability for fragments being detected as extensions of known transcripts, but with a larger value of D1 we could risk a failure to observe short transcripts near known transcripts. We used D2 to separate any two clusters on the same strand. Based on Ensembl genome annotation version 79, the mean of intronic lengths in the zebrafish genome is approximated 3 kb (standard deviation, SD 8.2 kb) and the mean of intragenic lengths is 64 kb (SD 112 kb). Since sizes of both intragenic and intronic regions vary to a high extent, it is difficult to choose a generic value suitable to all transcripts. The results we present were based on a setting of D2 to 5 kb (Fig 1B). However, we also tested a setting of D2 to 10 kb, which resulted in a slightly reduction of the total cluster number, from 60194 to 46376 (S5 Fig). The discovery of a larger number of putative NTRs (672 vs. 648) with the setting of D2 to 10 kb was due to less clusters with single fragments being removed (S5 Fig). Increasing D2 could cause fusion of two putative NTRs, while decreasing it might risk getting more incomplete NTRs. However, the selected settings of D2 to 5 kb worked well based on the outcome of both *in silico* and experimental validations.

### NTRs discovery and validation

We pooled RNA-Seq data from all three runs and four developmental stages to enhance detection of NTR at discovery step since they were all from early developmental stages, but we validated the presence of NTRs at individual developmental stage by independent experiments separately.

We excluded multiple mapped reads to avoid complexity they could introduce in both fragment formation and clustering steps, although by doing so we risked losing information. However, by using *in silico* and/or experimental validations the influence of multiple mapped reads on the structures of predicted NTRs could be compensated for. We excluded 2448 NTRs with only one fragment over 50 bp without junction observed (Fig 1B). These single fragment NTRs could be candidates for novel noncoding gene transcripts, however this is beyond the scope of this study. Moreover, we also excluded 1461 clusters containing only one splicing junction (Fig 1B). As consequence, the number of NTRs we report here is smaller compared to those from previous studies [7, 8, 12], however the NTRs discovered in the present study could be more reliable because they were subject to several filtering steps with stricter criteria. On the other hand, most previously reported NTRs also included a large number of isoforms for individual NTRs. Therefore, the numbers could be larger as well.

In this study, we randomly selected and successfully validated 4 NTRs by using RT-PCR. Interestingly, among these validated NTRs, 3 NTRs (Fig 3B, 3C and 3D) contained a very large intron-like structure in each, which was detected based on junction information and confirmed by validation. The large intron-like structure could split those NTRs into two parts by D2 without junction information. Therefore, the junction information was very valuable.

Furthermore, validation also rendered evidence of non-predicted transcripts with alternative splicing sites (Fig 3C and 3D). Further validations are needed to clarify boundaries at both 5’ and 3’ ends of the discovered NTRs.

### Regulation of discovered NTRs during development

In this study we have generated a list of NTRs that are putative candidate genes that may provide novel information on early embryo development, as well as being novel zebrafish genes with homology to human or other mammalian genes.

The majority of the discovered NTRs were expressed at low levels in early developmental stages (S1 Fig), which might be a reason for them remaining previously unannotated. However, when validating using qRT-PCR, all 10 randomly selected NTRs were detected, albeit at different expression levels (Fig 4B, S4 Fig). In addition, the expression patterns shown for the different NTRs were similar when comparing the gene expression profiles between RNA-Seq and qRT-PCR (Fig 4A and 4B). Furthermore, the expression profiles show that the majority of the investigated NTRs peak in expression levels at MBT or at 50% epiboly (post-MBT). (S1 and S2 Figs) This indicates that processes during MBT induce or activate the expression of the NTRs, although the majority of the 152 NTRs are present already at 1 cell stage (S1 and S2 Figs). It has been shown in previous studies that there is an overall increase in expression post-MBT [7, 8].

Transcripts originating from the zygote genome have been shown to encode for factors involved in biological processes such as differentiation, pattern formation and cell morphology among others and thus some of the 152 NTRs may fall into these categories [7, 8, 12]. For example, the expression of NTR57 measured by two platforms (RNA-Seq and qRT-PCR) showed a similar pattern, down regulated at 16-cell, upregulated at 512-cell stage, and further downregulated at 50% epiboly, suggesting that this NTR may be involved in MBT and deactivated after MBT. However, the standard error of NTR57 gene expression in 512-cell is very large among the biological replicates (Fig 4B). This standard error may reflect a biological variance between different individual female zebrafish, or it may reflect embryo developmental competence differences. Since the embryos were sampled at MBT (512-cell stage) it is not possible to get information on whether a high or low relative expression of NTR57 could be associated with any change in developmental competence or embryo survival past the 512-cell stage. It would be of particular interest to investigate the NTRs with relative high expression values, such as NTR157, NTR8, NTR61 and NTR94 in future studies. The zebrafish model system benefits from the continuous discovery of novel genes and more gene orthologues with candidate human disease genes. Although the putative biological relevance of the NTRs discovered is difficult to determine based only on the information obtained within this study, the validated transcripts give some indication on the biological relevance of the NTR candidates. Future studies will be needed in order to determine the function and characteristics of each of these.

## Conclusion

With the increasing number of RNA-Seq data from a large variety of species, there is a vast amount of novel information that can be found using relatively simple in-house bioinformatics pipelines. We here described the development of an in-house bioinformatics pipeline for NTR discovery based on RNA-Seq data sets. Using this pipeline we discovered 152 putative NTRs that had not been previously annotated. Four randomly selected NTRs were successfully validated experimentally using RT-PCR. Using qRT-PCR we showed that the expression levels of 10 NTRs varied during the four early developmental stages investigated, suggesting that these NTRs may have a specific role in zebrafish early development. However, the characterization and function of these NTRs was not within the scope of present study and will require further investigation.

The 152 discovered NTRs provide new information for zebrafish genome characterization and zebrafish developmental studies as well as new gene candidates for developmental studies, thus further increasing the value of the zebrafish model system for the scientific community.

## Supporting Information

S1 Fig Expression profiles of 152 NTRs in the four developmental stages. The expression levels in the four developmental stages were FPKM values of the biological replicates, scaled in each NTR when clustering all 152 NTRs.
(PDF)

S2 Fig Expression patterns of NTRs in the four developmental stages. The expression levels of the six selected patterns were obtained from the biological replicates. MBT stands for mid-blastula transition stage.
(PDF)

S3 Fig Primer loci of 10 randomly selected NTRs. (PDF)

S4 Fig Expression pattern of 10 validated NTRs in the four developmental stages. Three biological replicates of each NTR in each developmental stage were used for quantitative measurement with b-actin as control. ΔCt = Ct(NTR)-Ct(b-actin). Average -ΔCt values were applied to demonstrate the expression patterns in 10 validated NTRs.
(PDF)

S5 Fig Different settings of parameters D1 and D2. D1 is the distance between NTR and annotated genes, and D2 is the distance between two NTRs.
(PDF)

S1 Table Primers used in NTR structural validation and NTR expression profiling. (XLSX)

S2 Table 648 putative NTRs without annotation in Ensembl Genome Annotation Release 79. (XLSX)
