# Supplementary material for: Identification of Novel Transcribed Regions in Zebrafish (Danio rerio) Using RNA-Sequencing
Source: PLoS One. 2016 Jul 27;11(7):e0160197. doi: 10.1371/journal.pone.0160197 (PMC4962977; doi:10.1371/journal.pone.0160197)

S1 Fig. Expression profiles of 152 NTRs in the four developmental stages

The expression levels in the four developmental stages were FPKM values of the biological replicates, scaled in each NTR when clustering all 152 NTRs.

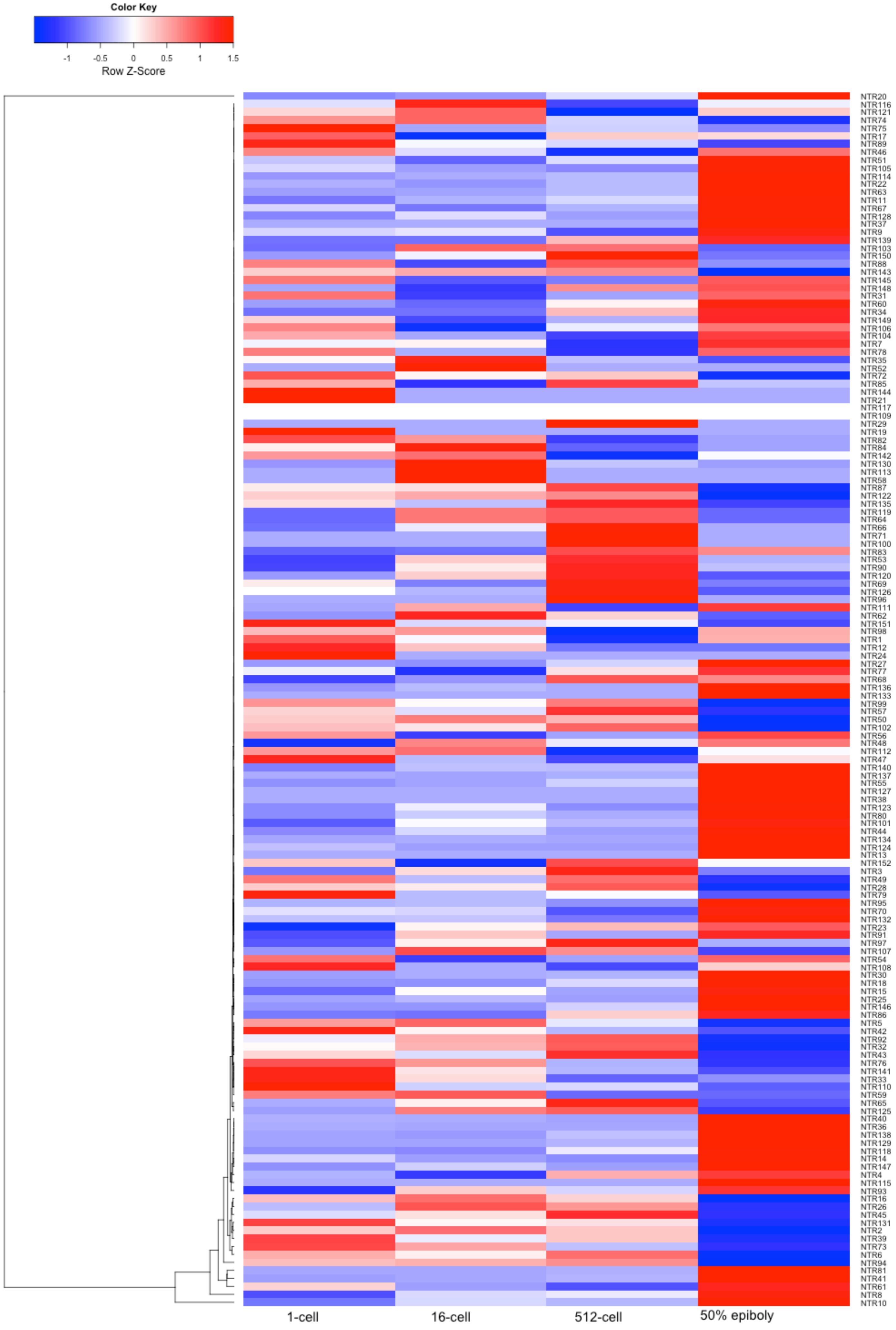

Supplement: S1 Fig — The expression levels in the four developmental stages were FPKM values of the biological replicates, scaled in each NTR when clustering all 152 NTRs. (PDF) [file pone.0160197.s001.pdf]
