# Supplementary material for: Identification of Novel Transcribed Regions in Zebrafish (Danio rerio) Using RNA-Sequencing
Source: PLoS One. 2016 Jul 27;11(7):e0160197. doi: 10.1371/journal.pone.0160197 (PMC4962977; doi:10.1371/journal.pone.0160197)

S2 Fig. Expression patterns of NTRs in the four developmental stages

The expression levels of the six selected patterns were obtained from the biological replicates. MBT stands for mid-blastula transition stage.

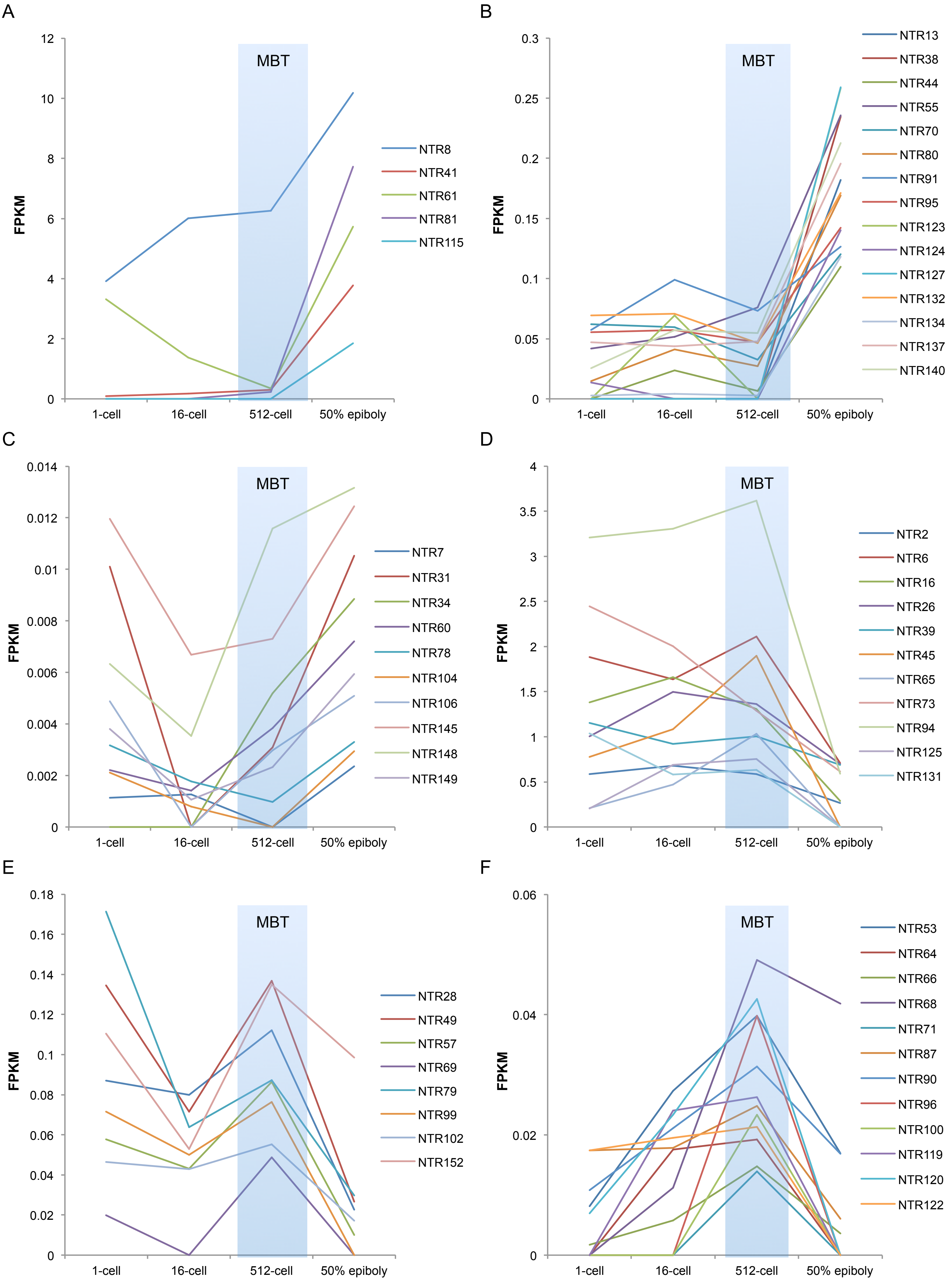

Supplement: S2 Fig — The expression levels of the six selected patterns were obtained from the biological replicates. MBT stands for mid-blastula transition stage. (PDF) [file pone.0160197.s002.pdf]
