# Supplementary figures and images for: Identification of Novel Transcribed Regions in Zebrafish (Danio rerio) Using RNA-Sequencing
Source: PLoS One. 2016 Jul 27;11(7):e0160197. doi: 10.1371/journal.pone.0160197 (PMC4962977; doi:10.1371/journal.pone.0160197)

S3 Fig. Primer loci of 10 randomly selected NTRs

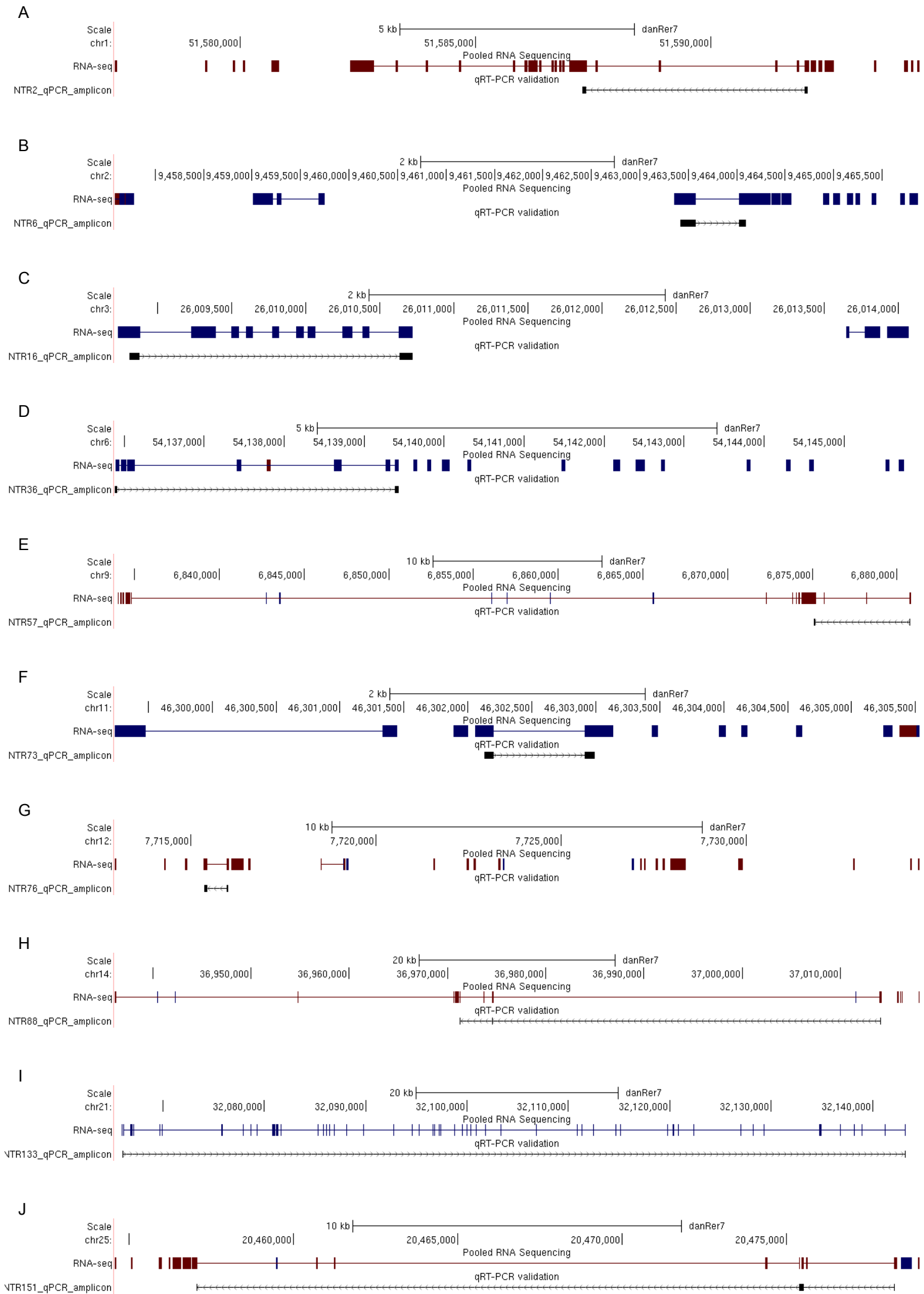

Supplement: S3 Fig — (PDF) [file pone.0160197.s003.pdf]
