# Supplementary material for: Identification of Novel Transcribed Regions in Zebrafish (Danio rerio) Using RNA-Sequencing
Source: PLoS One. 2016 Jul 27;11(7):e0160197. doi: 10.1371/journal.pone.0160197 (PMC4962977; doi:10.1371/journal.pone.0160197)

S4 Fig. Expression pattern of 10 validated NTRs in the four developmental stages

Three biological replicates of each NTR in each developmental stage were used for quantitative measurement with b-actin as control. ΔCt = Ct(NTR)-Ct(b-actin). Average -ΔCt values were applied to demonstrate the expression patterns in 10 validated NTRs.

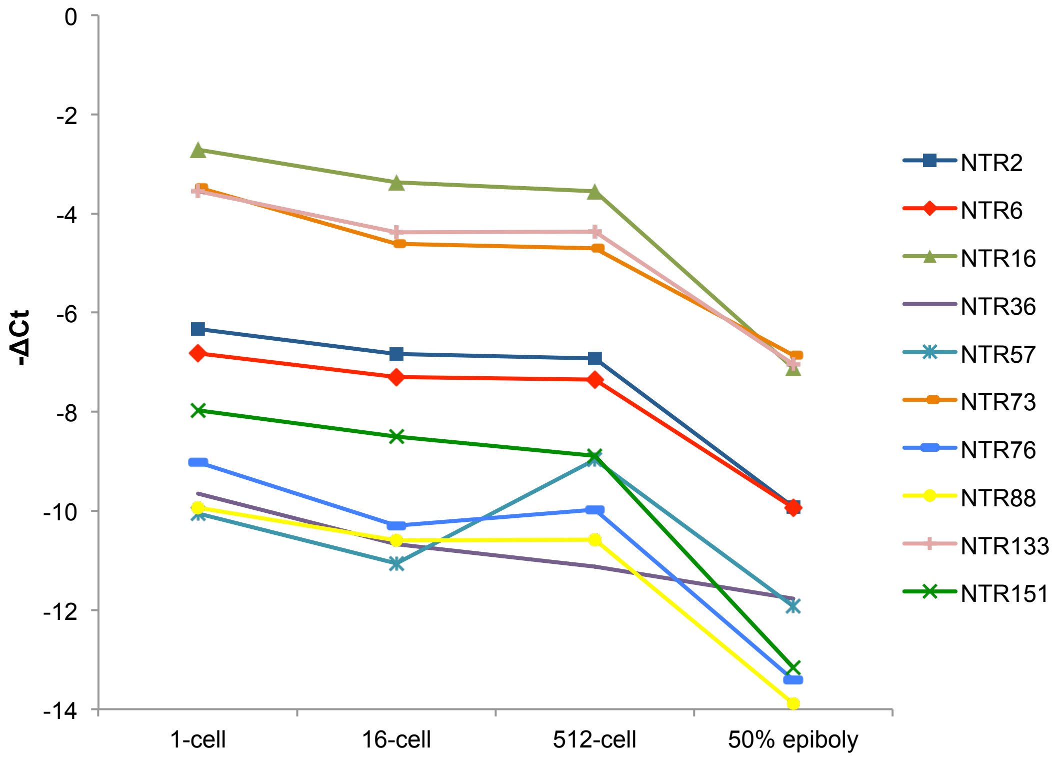

Supplement: S4 Fig — Three biological replicates of each NTR in each developmental stage were used for quantitative measurement with b-actin as control. ΔCt = Ct(NTR)-Ct(b-actin). Average -ΔCt values were applied to demonstrate the expression patterns in 10 validated NTRs. (PDF) [file pone.0160197.s004.pdf]
