# Supplementary material for: Identification of Novel Transcribed Regions in Zebrafish (Danio rerio) Using RNA-Sequencing
Source: PLoS One. 2016 Jul 27;11(7):e0160197. doi: 10.1371/journal.pone.0160197 (PMC4962977; doi:10.1371/journal.pone.0160197)

**S5 Fig. Different settings of parameters D1 and D2**

D1 is the distance between NTR and annotated genes, and D2 is the distance between two NTRs.

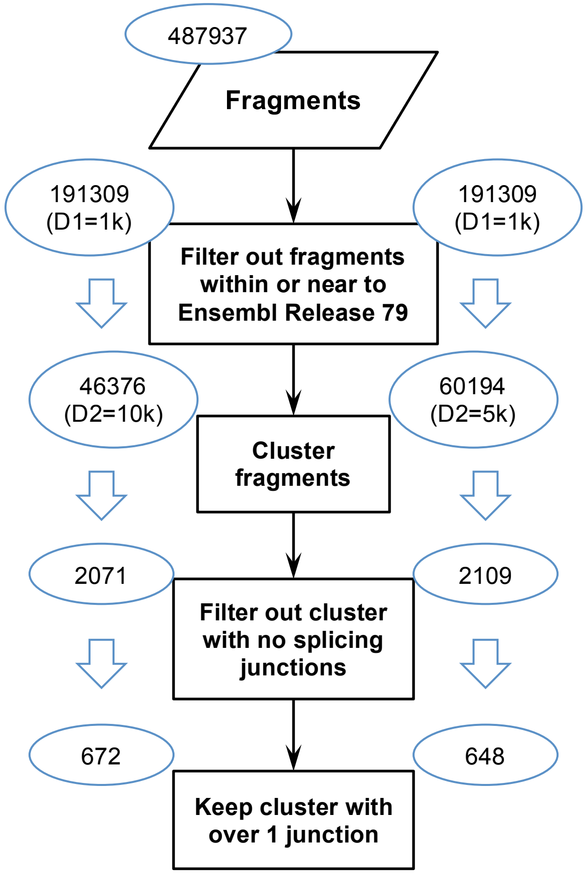

Supplement: S5 Fig — D1 is the distance between NTR and annotated genes, and D2 is the distance between two NTRs. (PDF) [file pone.0160197.s005.pdf]
